# The complete chloroplast genome of *Phyllostachys glauca* (Bambusoideae), a dominant bamboo species in limestone mountains endemic to China

**DOI:** 10.1080/23802359.2020.1810152

**Published:** 2020-08-25

**Authors:** Binbin Cao, Tingting Ge, Shixiong Ding, Chunce Guo

**Affiliations:** Jiangxi Provincial Key Laboratory for Bamboo Germplasm Resources and Utilization, Forestry College, Jiangxi Agricultural University, Nanchang, P. R. China

**Keywords:** Bambusoideae Arundinarieae, *Phyllostachys glauca*, phylogenetic relationship

## Abstract

*Phyllostachys glauca* is a dominant species in limestone mountains endemic to China. Here, we characterized its complete chloroplast genome. It is a circular DNA molecule of 139,689 bp in length, including a pair of 21,798 bp inverted repeats (IRs), a 12,872 bp small single-copy (SSC) region and an 83221 bp large single-copy (LSC) region. The total GC content of *P. glauca* chloroplast genome was 38.9%, and it encodes a total of 137 functional genes, including 89 protein-coding genes, 40 tRNA genes, and 8 rRNA genes. The phylogenetic analysis shows that *P. glauca* is highly clustered in the *Phyllostachys* clade (V), sister to the lineage of *P. nigra* var. *henonis* + *P. sulphurea*.

Limestone area is usually ecologically fragile complex terrains, where the topography considerably affects vegetation coverage (Pu et al. 2017). Adapted to drought stress by regulating the concentration and distribution of nutrient elements, *Phyllostachys glauca*, a temperate woody bamboo, is one of the dominant species in limestone mountains of southern China (Du et al. 1994; Liang et al. 2019). Like most bamboo plants, *P. glauca* shows a pattern of massive synchronized flowering and leads to large-scale death after flowering (Zheng et al. 2020).

In this study, we obtained and reported the complete chloroplast genome of *P. glauca*. Fresh and young leaves as sequencing materials were collected from the bamboo garden of Jiangxi Agricultural University, China (28°45′40″N, 115°49′31″E). The voucher specimen (JXAU-20201532) was deposited at the herbarium of the College of Forestry, Jiangxi Agricultural University, China. Illumina paired-end (PE) library was prepared and sequenced in the Nanjing Novogene Bio-technology Co., Ltd., China. The clean reads were assembled by using GetOrganelle v1.5 (Jin et al. 2018). Subsequently, the plastome was annotated using GeSeq (Tillich et al. 2017) and Geneious 9.0.5 (http://www.geneious.com/), and detected simple sequence repeats (SSR) by MISA (http://pgrc.ipk-gatersleben.de/misa). The annotated complete chloroplast genome of *P. glauca* was deposited in GenBank under the accession no. MT657329.

The chloroplast genome of *P. glauca* is a typical circular structure of 139,689 bp in length, and consisting of a large single-copy region with 83,221 bp (LSC), a small single-copy region with 12,872 bp (SSC), and two inverted repeat regions with 21,798 bp (IRs). The total GC content of the chloroplast genome is 38.9%, meanwhile the corresponding value of LSC, SSC, and IR regions is 37.0%, 33.2% and 44.2%, respectively. The whole genome contained 137 functional genes, including 89 protein-coding genes, 40 tRNA genes, and 8 rRNA genes.

To evaluate the phylogenetic position of *P. glauca*, additional 30 complete chloroplast genomes in the trib. Arundinarieae, together with three species as outgroup, were retrieved from NCBI (Figure 1). All sequences were aligned with MAFFT 7.409 (Katoh and Toh 2010). Maximum-likelihood tree and Bayes tree were constructed using RAxML 8.2.8 (Stamatakis 2014) and MrBayes 3.2.6 (Ronquist and Huelsenbeck 2003), respectively. The results showed that *P. glauca* is highly clustered in the *Phyllostachys* clade (V), as a sister group with the lineage of *P. nigra* var. *henonis* and *P. sulphurea*. In addition, the relationships within *Phyllostachys* genus are still weakly supported in the phylogenetic tree with short internodes, reflecting the probable recent rapid radiation during the evolution.

**Figure 1.**
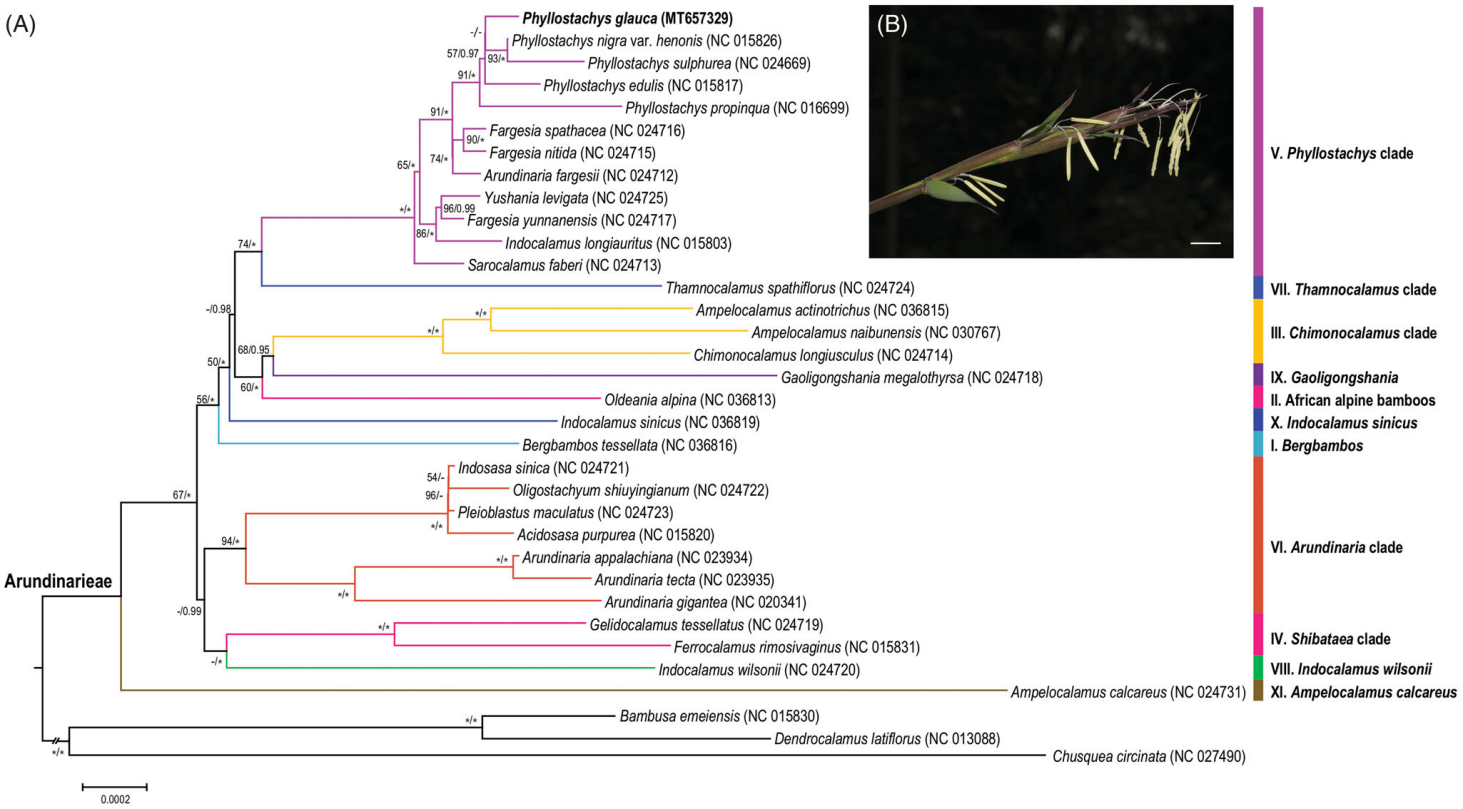
Phylogenetic position and flowers of *P. glauca*. A. Maximum likelihood tree based on protein coding sequences of chloroplast genomes from 34 bamboos. Numbers associated with branches are ML bootstrap values, and Bayesian posterior probabilities, respectively. ‘*’ indicate 100% bootstrap support or 1.0 posterior probability. ‘-’ indicate the bootstrap support or posterior probability lower than 50% or 0.5. B. Flowering branches of *P. glauca*, showing mixed inflorescence. Scale bar = 10 mm.

## Data Availability

The data that support the findings of this study are available in ‘Dryad’ at http://doi:10.5061/dryad.m37pvmd07, reference number [m37pvmd07].
